# Direct Whole-Genome Sequencing of Cutaneous Strains of *Haemophilus ducreyi*

**DOI:** 10.3201/eid2404.171726

**Published:** 2018-04

**Authors:** Michael Marks, Maria Fookes, Josef Wagner, Rosanna Ghinai, Oliver Sokana, Yaw-Adu Sarkodie, Anthony W. Solomon, David C.W. Mabey, Nicholas R. Thomson

**Affiliations:** London School of Hygiene & Tropical Medicine, London, UK (M. Marks, R. Ghinai, A.W. Solomon, D.C.W. Mabey, N.R. Thomson);; Hospital for Tropical Diseases, London (M. Marks, A.W. Solomon, D.C.W. Mabey);; Wellcome Trust Sanger Centre, Cambridge, UK (M. Fookes, J. Wagner, N.R. Thomson);; Solomon Islands Ministry of Health and Medical Services, Honiara, Solomon Islands (O. Sokana);; Kwame Nkrumah University of Science and Technology, Kumasi, Ghana (Y.-A. Sarkodie)

**Keywords:** Whole-genome sequencing, 16S rRNA gene sequencing, next-generation sequencing, yaws, chancroid, *Haemophilus ducreyi*, *Treponema pallidum*, children, skin disease, bacteria, Solomon Islands, Ghana

## Abstract

*Haemophilus ducreyi,* which causes chancroid, has emerged as a cause of pediatric skin disease. Isolation of *H. ducreyi* in low-income settings is challenging, limiting phylogenetic investigation. Next-generation sequencing demonstrates that cutaneous strains arise from class I and II *H. ducreyi* clades and that class II may represent a distinct subspecies.

Since 2000, the global prevalence of chancroid, caused by *Haemophilus ducreyi*, has declined (*1*). *H. ducreyi* is an emerging cause of cutaneous ulcers in tropical countries (*1*–*4*). Cutaneous lesions of *H. ducreyi* are difficult to distinguish from other common causes of ulcerative skin disease, such as yaws (*3*,*4*), which presents problems in diagnosing yaws and has resulted in the World Health Organization recommending molecular testing of yaws-like lesions (*5*).

Culturing *H. ducreyi* is challenging. PCR is usually used for diagnosis (*6*). Culture requirements limit sequencing and phylogenetic analyses. Traditional phylogenies divide genital strains of *H. ducreyi* into class I and II clades. Most studies suggest that cutaneous strains of *H. ducreyi* have diversified from within the class I clade (*7*,*8*), and a recent study reported cutaneous strains that appear to arise from class II strains (*9*). These studies have been limited by the number and geographic spread of samples included.

Next-generation sequencing enables whole-genome sequencing from clinical samples without prior culture, bypassing the culture requirements of *H. ducreyi* and enabling more detailed phylogenetic analysis. We performed next-generation sequencing on samples collected in previous surveys conducted in the Solomon Islands (in 2013) and Ghana (in 2014) (*2*,*4*). In both surveys, skin swab specimens had been collected from persons with chronic ulcerative lesions believed, at the time, to be clinically consistent with yaws. DNA was prepared for the current study from samples with residual material from those original surveys. The London School of Hygiene & Tropical Medicine, Solomon Islands National Health Research, and Kwame Nkrumah University of Science and Technology ethics committees approved these studies.

## The Study

**Figure 1:**
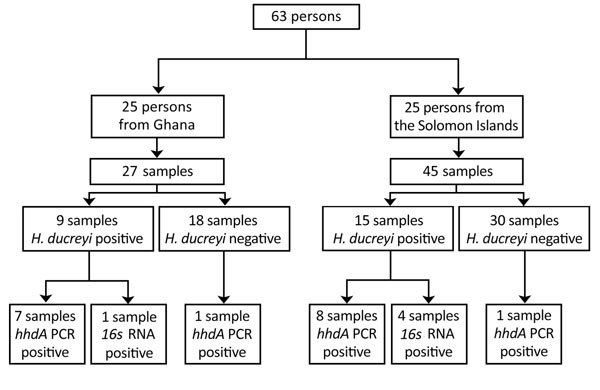
Flowchart of whole-genome sequencing of *Haemophilus ducreyi*. Samples were originally collected in 2 studies conducted in Ghana (2014) and the Solomon Islands (2013) (*2*,*4*). Results of the *H. ducreyi* PCR conducted in the original studies and of the 2 *H. ducreyi* PCRs performed in this study are shown.

We tested 72 samples from 63 persons (Figure 1). Twenty-five persons (27 samples) had been recruited in Ghana and 38 persons (45 samples) in the Solomon Islands. Median age of participants in the original studies was 9 years (interquartile range 7–11 years); 36 (57.1%) were male. In the original studies, 24 samples had tested positive for *H. ducreyi* using a 16S rRNA-targeted PCR (*2*,*4*): 15 from the Solomon Islands and 9 from Ghana.

In Ghana, samples were collected directly onto dry Dacron swabs. In the Solomon Islands, swab exudate was placed into transport medium (AssayAssure; Sierra Molecular, Incline Village, NV, USA) or onto an FTA Elute Card (Thermo-Fisher Scientific, Waltham, MA, USA). Samples were frozen at −20°C and shipped to the Centers for Disease Control and Prevention (Atlanta, GA, USA) on dry ice for the original laboratory analyses, which included real-time PCR for *Trepomema pallidum* subspecies *pertenue* (*7*) and a real-time 16S rRNA-targeted PCR for *H. ducreyi* (*2*,*4*). After testing, samples were shipped on dry ice to the London School of Hygiene & Tropical Medicine (London, UK) and frozen at −20°C before analysis.

We extracted DNA from residual sample material using QIAamp Mini kits (QIAGEN, Hilden, Germany) **(**Technical Appendix 1**)**. We screened DNA using a quantitative PCR (qPCR) targeting the *hhdA* gene and 16S rRNA gene sequencing for *H. ducreyi* (*6*,*10*). From samples that tested positive, we selected those with genomic DNA concentration >10 copies/μL for direct (non–culture-based) sequencing.

Genomic DNA was fragmented to an average size of 150 bp and subjected to DNA library creation using established Illumina paired-end protocols (*11*). We amplified adaptor-ligated libraries and indexed them by PCR. We used a portion of each library to create an equimolar pool and hybridized each pool to custom-made SureSelect RNA baits (Agilent Technologies, Santa Clara, CA, USA; baits based on published sequences of *H. ducreyi* [*12*]) (Technical Appendix 1). Targets were captured and amplified in accordance with manufacturer’s recommendations. We subjected enriched libraries to standard 75-bp end sequencing (HiSeq 2000; Illumina, San Diego, CA, USA). Samples’ public accession numbers are listed in Technical Appendix 2 Table 1). We used whole-genome sequence data to estimate phylogenies for *H. ducreyi* (Technical Appendix 1), including publicly available *H. ducreyi* genomes alongside those obtained in this study.

We identified *H. ducreyi* in 17 samples by *hhdA*-targeted qPCR and in 5 additional samples using an assay targeting the rRNA gene. From these 22 positive samples, we obtained 21 (95.5%) complete genomes from 13 persons from the Solomon Islands and 8 from Ghana. Mean coverage of *H. ducreyi* genomes was 91% (Technical Appendix 2 Table 1). We found no evidence of sequence heterozygosity that would indicate any participant was infected with multiple distinct strains of *H. ducreyi*.

**Figure 2:**
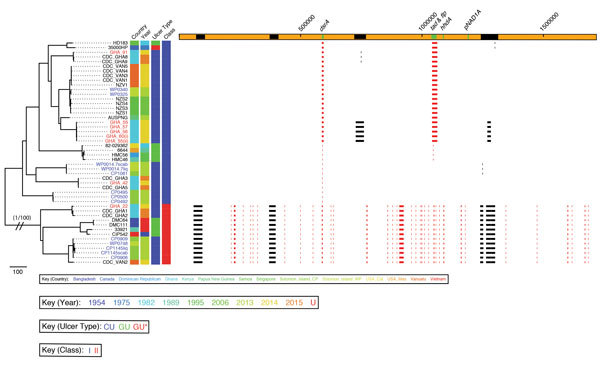
Phylogenetic tree of *Haemophilus ducreyi* genome sequences inferred from mapping using the *H. ducreyi* 35000HP strain as reference and after removing high-density single-nucleotide polymorphisms regions with Gubbins (*3*). Included are published genomes (black text), Ghanaian strains (gray text, GHA designations), and Solomon Islands strains (gray text, CP/WP designations). Sequences from cutaneous ulcers in Ghana and the Solomon Islands were found within both previously described clades of *H. ducreyi* class I and class II. Scale bar indicates nucleotide substitutions per site. An expanded version of this figure providing complete phylogeny details, including countries of origin, years, ulcer types, and genome region designations, is provided in Technical Appendix 1 Figure.

*H. ducreyi* sequences fell into both previously defined *H. ducreyi* clades: class I and class II. To estimate genetic distance between strains, we determined the number of single-nucleotide polymorphisms (SNPs) in pairwise whole-genome comparisons. The average distance between class I and class II sequences was 21,238 SNPs, compared with a maximum pairwise distance of 641 SNPs between class I sequences. We detected 4 major recombination blocks within class I genomes. These regions included the *dsrA*, *tad,* and *flp* loci, associated with serum resistance, tight adhesion, and production of fimbriae, respectively, functions important in micro-colony formation and potentially associated with virulence (Technical Appendix 1 Figure 1; Technical Appendix 2 Table 2) (*13*). The other regions of likely recombination were related to integrated prophage elements, implying *H. ducreyi* has an actively exchanging bacteriophage repertoire in its genome (Technical Appendix 1 Figure 1). These prophage elements included the region coding for the ctdABC genes, which have been associated with virulence (*14*). The class I prophage elements were absent from class II genomes but intermittently present in class I genomes (Figure 2). The presence or absence of the ctdABC coding region was not associated with cutaneous or genital ulcer disease. Another recombination region spanned the *hhdA* specific qPCR primer binding site. Samples with high sequence variation in this region tested negative for *H. ducreyi* by qPCR but gave high numbers of reads by 16S rRNA gene sequencing.

## Conclusions

We obtained whole-genome sequences of *H. ducreyi* without prior culture. Most earlier studies have suggested that cutaneous strains emerged by diversification from within the class I clade (*7*,*8*), although 1 study found, in keeping with our findings, cutaneous strains emerging from class II (*9*). We found genital and cutaneous strains are represented in all lineages of the expanded phylogenetic tree (*7*). We found considerable genetic variation between class I and class II *H. ducreyi* sequences. Together with existing 16S rRNA data and multilocus sequence typing data (*12*) these findings suggest class II strains might represent a discrete subspecies of *H. ducreyi*.

We identified 2 samples that had been negative in the original studies but were found to contain *H. ducreyi* DNA in the current study. Repeated freeze–thaw cycles and limited residual DNA volumes might have contributed to our lack of detection of *H. ducreyi* DNA in 4 samples that tested positive in the original studies (Figure 1). Five samples that returned a weak signal by *hhdA* qPCR contained class II clade *H. ducreyi* genomes. The failure of qPCR to detect *H. ducreyi* in these samples was most likely explained by variation in the sequence of the *hhdA* pPCR primer binding sites (*13*) between class I and II genomes (Figure 2), demonstrating our limited understanding of the diversity of these pathogens.

Culture for *H. ducreyi* is not practical in the low-income settings where cutaneous strains of this organism are endemic. Next-generation sequencing circumvents this problem by enabling whole-genome sequencing direct from clinical samples. This approach considerably strengthens our ability to sequence *H. ducreyi* and broaden knowledge of this emerging pathogen.

Technical Appendix 1 Additional methods; phylogenetic tree of *Haemophilus ducreyi* genomes; and full diagram of *H. ducreyi* genome sequence coverage.

Technical Appendix 2 Illumina sequenced genomes of *Haemophilus ducreyi* and high-density single-nucleotide polymorphism regions identified and removed for the *H. ducreyi* phylogeny.

## References

[1] González-Beiras C, Marks M, Chen CY, Roberts S, Mitjà O. Epidemiology of *Haemophilus ducreyi* infections. Emerg Infect Dis. 2016;22:1–8. 10.3201/eid2201.150425 26694983 PMC4696685

[2] Ghinai R, El-Duah P, Chi K-H, Pillay A, Solomon AW, Bailey RL, et al. A cross-sectional study of ‘yaws’ in districts of Ghana which have previously undertaken azithromycin mass drug administration for trachoma control. PLoS Negl Trop Dis. 2015;9:e0003496. 10.1371/journal.pntd.0003496 25632942 PMC4310597

[3] Mitjà O, Lukehart SA, Pokowas G, Moses P, Kapa A, Godornes C, et al. *Haemophilus ducreyi* as a cause of skin ulcers in children from a yaws-endemic area of Papua New Guinea: a prospective cohort study. Lancet Glob Health. 2014;2:e235–41. 10.1016/S2214-109X(14)70019-1 25103064

[4] Marks M, Chi K-H, Vahi V, Pillay A, Sokana O, Pavluck A, et al. *Haemophilus ducreyi* associated with skin ulcers among children, Solomon Islands. Emerg Infect Dis. 2014;20:1705–7. 10.3201/eid2010.140573 25271477 PMC4193279

[5] Marks M, Mitjà O, Vestergaard LS, Pillay A, Knauf S, Chen C-Y, et al. Challenges and key research questions for yaws eradication. Lancet Infect Dis. 2015;15:1220–5. 10.1016/S1473-3099(15)00136-X 26362174 PMC4668588

[6] Chen C-Y, Ballard RC. The molecular diagnosis of sexually transmitted genital ulcer disease. In: MacKenzie CR, Henrich, editors. Diagnosis of sexually transmitted diseases—methods and protocols. New York: Springer-Verlag; 2012. p. 103–12. 10.1007/978-1-61779-937-2_6 22782813

[7] Gangaiah D, Webb KM, Humphreys TL, Fortney KR, Toh E, Tai A, et al. *Haemophilus ducreyi* cutaneous ulcer strains are nearly identical to Class I genital ylcer strains. PLoS Negl Trop Dis. 2015;9:e0003918. 10.1371/journal.pntd.0003918 26147869 PMC4492979

[8] Gangaiah D, Marinov GK, Roberts SA, Robson J, Spinola SM. Draft whole-genome sequence of *Haemophilus ducreyi* strain AUSPNG1, isolated from a cutaneous ulcer of a child from Papua New Guinea. Genome Announc. 2016;4:e01661–15. 10.1128/genomeA.01661-15 26847887 PMC4742684

[9] Gangaiah D, Spinola SM. *Haemophilus ducreyi* cutaneous ulcer strains diverged from both Class I and Class II genital ulcer strains: implications for epidemiological studies. PLoS Negl Trop Dis. 2016;10:e0005259. 10.1371/journal.pntd.0005259 28027326 PMC5222509

[10] Marks M, Vahi V, Sokana O, Puiahi E, Pavluck A, Zhang Z, et al. Mapping the epidemiology of yaws in the Solomon Islands: a cluster randomized survey. Am J Trop Med Hyg. 2015;92:129–33. 10.4269/ajtmh.14-0438 25422395 PMC4347367

[11] Orle KA, Gates CA, Martin DH, Body BA, Weiss JB. Simultaneous PCR detection of *Haemophilus ducreyi, Treponema pallidum*, and herpes simplex virus types 1 and 2 from genital ulcers. J Clin Microbiol. 1996;34:49–54. 8748271 10.1128/jcm.34.1.49-54.1996 PMC228728

[12] Quail MA, Kozarewa I, Smith F, Scally A, Stephens PJ, Durbin R, et al. A large genome center’s improvements to the Illumina sequencing system. Nat Methods. 2008;5:1005–10. 10.1038/nmeth.1270 19034268 PMC2610436

[13] Ricotta EE, Wang N, Cutler R, Lawrence JG, Humphreys TL. Rapid divergence of two classes of *Haemophilus ducreyi.* J Bacteriol. 2011;193:2941–7. 10.1128/JB.01400-10 21515774 PMC3133207

[14] Janowicz DM, Cooney SA, Walsh J, Baker B, Katz BP, Fortney KR, et al. Expression of the Flp proteins by *Haemophilus ducreyi* is necessary for virulence in human volunteers. BMC Microbiol. 2011;11:208. 10.1186/1471-2180-11-208 21939541 PMC3201912

