# Supplementary material for: Direct Whole-Genome Sequencing of Cutaneous Strains of Haemophilus ducreyi
Source: Emerg Infect Dis. 2018 Apr;24(4):786–9. doi: 10.3201/eid2404.171726 (PMC5875288; doi:10.3201/eid2404.171726)
Supplement: Technical Appendix 1 — Additional methods; phylogenetic tree of Haemophilus ducreyi genomes; and full diagram of H. ducreyi genome sequence coverage. [file 17-1726-Techapp-s1.pdf]

Article DOI: https://doi.org/10.3201/eid2404.171726

Direct Whole-Genome Sequencing of Cutaneous Strains of *Haemophilus ducreyi*

Technical Appendix 1

DNA Extraction Methods Used

Dry Dacron swab tips from Ghana were suspended in 1 mL of lysis buffer (10 mM Tris, pH 8.0; 0.1M EDTA, pH 8.0; 0.5% sodium dodecyl sulphate), vortexed vigorously for 5 min, and the supernatants divided into 3 tubes. The Qiagen (Hilden, Germany) buccal swab spin protocol (which includes a proteinase K reaction step) was then followed. The same procedure was applied to Solomon Islands samples (transported in AssayAssure transport buffer, Sierra Molecular, Incline Village, USA); lesion crusts were suspended in the liquid buffer and pelleted, and DNA extractions were performed on both the liquid phase (as above) and pellet. DNA extraction of the lesion pellet was performed as follows: pellets were resuspended in 500 mL of lysis buffer, treated with proteinase K, and transferred to a Lysing Matrix E tube (MP Biomedicals, Santa Ana, CA, USA) prefilled with various sized glass beads. 200 mL of AL buffer was added to this. Filled tubes were homogenized for 40 sec at speed 6,000 rpm (FastPrep Homogenizer, MP Biomedicals). The tubes were then centrifuged for 10 min at 13,200 rpm and the Qiagen DNA extraction protocol followed as above. The DNA was then extracted according to manufacturers' instructions. Each DNA sample was eluted in 150 μL AE buffer.

SureSelect Probe Design

SureSelect probe design was based on str. 35000HP (EMBL accession no. AE017143). Agilent 120 bp RNA baits were designed to cover the pan-genome consensus sequence to a depth of 3×. RNA baits were screened against the pan-genome consensus sequence for redundancy (see http://earray.chem.agilent.com/suredesign). All captured DNA was validated by sequencing and mapping to an appropriate reference genome.

Bio-informatics Pipeline

Reads were mapped (*1*) using SMALT to the reference genomes *of H. ducreyi* strain 35000HP (GenBank accession no. NC_002940), respectively. Whole-genome single nucleotide polymorphisms (SNPs) were identified using SamTools (*2*). Gubbins was used to identify recombination blocks (defined by having high SNP density) using the whole-genome SNP data of each sample as previously described (*3*). SNPs in these recombination blocks were excluded from phylogenetic analysis because they do not represent the underlying phylogeny of the host (Technical Appendix 1 Figure 2; Technical Appendix 2 Table 2, https://wwwnc.cdc.gov/EID/article/24/4/17-1726-Techapp2.xlsx). The remaining chromosomal SNP alleles from each isolate were concatenated to generate a multiple alignment of all SNPs, per isolate. Maximum likelihood phylogenetic trees were estimated from these remaining SNPs using RAxML with a general time reversible site model with gamma correction for among-site variation and with 100 bootstrap replicates (*4*). De novo genome assemblies were performed as previously described (*5*) or using SPAdes (*6*). Contigs were automatically annotated using in-house pipelines. Genes of interest were identified and curated by hand in pairwise comparisons with the appropriate reference genome using the Artemis Comparison Tool or Artemis (*7*). Basic mapping and assembly statistics are listed in re listed in Technical Appendix 2 Table 1 (https://wwwnc.cdc.gov/EID/article/24/4/17-1726-Techapp2.xlsx) , including the in silico simulated reassemblies of the 2 reference sequences to determine the achievable reassembly size from using Illumina 75-bp paired-end reads and the standard assembly protocols used in this study on the simulated reference sequence data.

References

1. Sanger Institute. SMALT [cited 2016 Dec 20]. http://www.sanger.ac.uk/science/tools/smalt-0

2. Li H, Handsaker B, Wysoker A, Fennell T, Ruan J, Homer N, et al.; 1000 Genome Project Data Processing Subgroup. The Sequence Alignment/Map format and SAMtools. Bioinformatics. 2009;25:2078–9. PubMed

3. Croucher NJ, Page AJ, Connor TR, Delaney AJ, Keane JA, Bentley SD, et al. Rapid phylogenetic analysis of large samples of recombinant bacterial whole genome sequences using Gubbins. Nucleic Acids Res. 2015;43:e15. PubMed

4. Stamatakis A. RAxML-VI-HPC: maximum likelihood-based phylogenetic analyses with thousands of taxa and mixed models. Bioinformatics. 2006;22:2688–90. PubMed

5. Bronowski C, Fookes MC, Gilderthorp R, Ashelford KE, Harris SR, Phiri A, et al. Genomic characterisation of invasive non-typhoidal *Salmonella enterica* subspecies *enterica* serovar Bovismorbificans isolates from Malawi. PLoS Negl Trop Dis. 2013;7:e2557. PubMed

6. Bankevich A, Nurk S, Antipov D, Gurevich AA, Dvorkin M, Kulikov AS, et al. SPAdes: a new genome assembly algorithm and its applications to single-cell sequencing. J Comput Biol. 2012;19:455–77. PubMed

7. Carver T, Berriman M, Tivey A, Patel C, Böhme U, Barrell BG, et al. Artemis and ACT: viewing, annotating and comparing sequences stored in a relational database. Bioinformatics. 2008;24:2672–6. PubMed

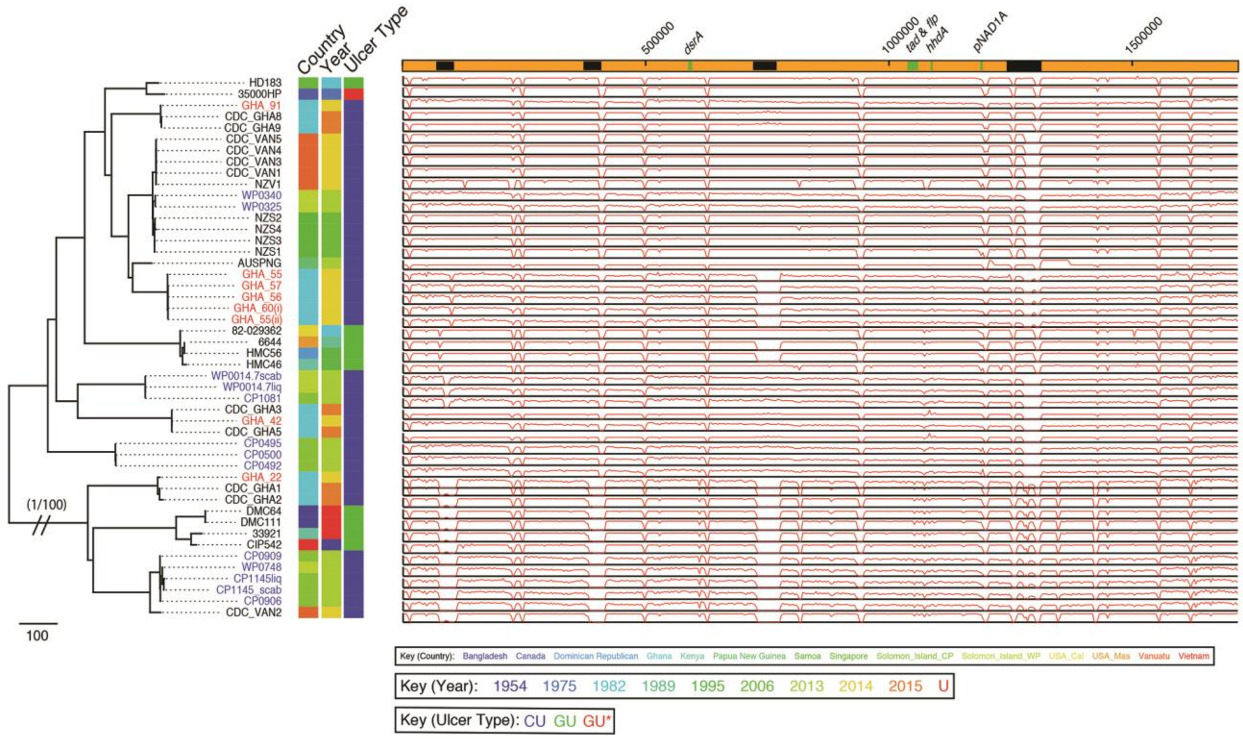

Technical Appendix 1 Figure 1. Phylogenetic tree of *Haemophilus ducreyi* genomes. Phylogenetic tree of *H. ducreyi* sequences inferred from mapping using the *H. ducreyi* 35000HP strain as reference and after removing high-density single-nucleotide polymorphisms regions with Gubbins (*3*). These regions are shown in the main frame below the genome with red for those events shared by ≥1 strains and blue for

those unique to a single strain. Included are published genomes (black labels), Ghanaian strains (red labels), and Solomon Islands strains (blue labels). Over the genome (orange bar), prophage-related regions are black and other relevant regions are green and labeled. Coordinates are in bps with respect to the reference. Sequences from cutaneous ulcers in Ghana and the Solomon Islands were found within both previously described clades of *H. ducreyi* Class I and Class II. Scale bar indicates nucleotide substitutions per site.

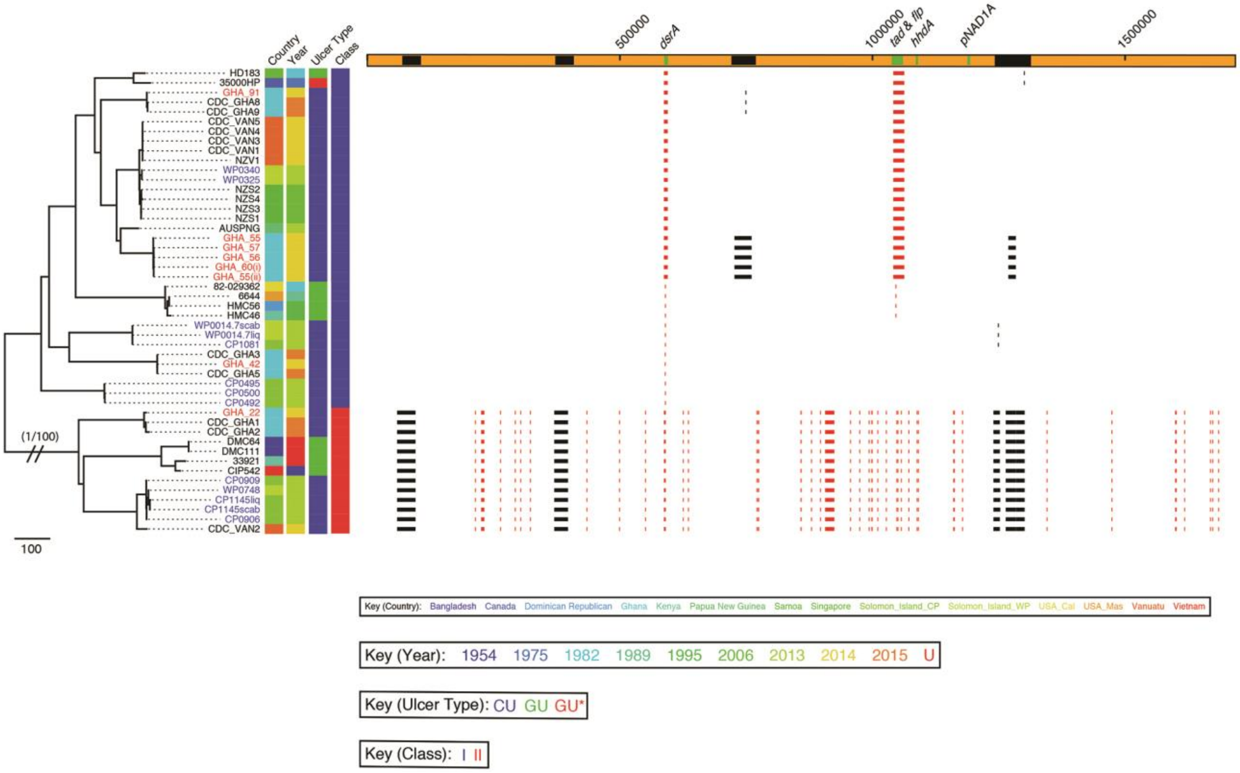

Technical Appendix 1 Figure 2. *Haemophilus ducreyi* genome sequence coverage. Coverage of genomes mapped to the reference 35000HP strain. Over the genome, phage locations are in black. Class I prophage elements were absent from Class II genomes but intermittently present in Class I genomes. Scale bar indicates nucleotide substitutions per site.
